# Mechanism of Mitochondrial Transcription Factor A Attenuation of CpG-Induced Antibody Production

**DOI:** 10.1371/journal.pone.0157157

**Published:** 2016-06-09

**Authors:** Christopher S. Malarkey, Claire E. Gustafson, Jessica F. Saifee, Raul M. Torres, Mair E. A. Churchill, Edward N. Janoff

**Affiliations:** 1 Department of Pharmacology and the Program in Structural Biology and Biochemistry, University of Colorado School of Medicine, Aurora, CO, 80045, United States of America; 2 Mucosal and Vaccine Research Program Colorado (MAVRC), Department of Medicine and the Program in Immunology, University of Colorado School of Medicine, Aurora, CO, 80045, United States of America, and Denver Veterans Affairs Medical Center, Denver, CO, 80220, United States of America; 3 Department of Immunology and Microbiology, University of Colorado School of Medicine, Aurora, CO, 80045, United States of America; Florida International University Bimolecular Sciences Institute, UNITED STATES

## Abstract

Mitochondrial transcription factor A (TFAM) had previously been shown to act as a damage associated molecular pattern with the ability to enhance CpG-A phosphorothioate oligodeoxynucleotide (ODN)-mediated stimulation of IFNα production from human plasmacytoid dendritic cells. Examination of the mechanism by which TFAM might influence CpG ODN mediated innate immune responses revealed that TFAM binds directly, tightly and selectively to the structurally related CpG-A, -B, and -C ODN. TFAM also modulated the ability of the CpG-B or -C to stimulate the production of antibodies from human B cells. TFAM showed a dose-dependent modulation of CpG-B, and -C -induced antibody production from human B cells *in vitro*, with enhancement of high dose and inhibition of low doses of CpG stimulation. This effect was linked to the ability of TFAM to directly inhibit the binding of CpG ODNs to B cells, in a manner consistent with the relative binding affinities of TFAM for the ODNs. These data suggest that TFAM alters the free concentration of the CpG available to stimulate B cells by sequestering this ODN in a TFAM-CpG complex. Thus, TFAM has the potential to decrease the pathogenic consequences of exposure to natural CpG-like hypomethylated DNA *in vivo*, as well as such as that found in traumatic injury, infection, autoimmune disease and during pregnancy.

## Introduction

The ability of the innate immune system to protect against and respond appropriately to pathogens and inflammatory stimuli is dependent on an elaborate set of pattern recognition receptors and downstream signaling events [1]. As effectors of innate immune responses, toll-like receptors (TLRs) recognize specific motifs found in pathogen-derived and self-antigens. One such motif is derived from bacterial, fetal and mitochondrial DNA [2, 3]. This DNA is generally hypomethylated and harbors particular cytosine-phosphate-guanosine (CpG) sequences. CpG oligodeoxynucleotides (ODNs) engage cell surface receptors, such as the receptor for Advanced Glycation Endproducts (RAGE) [4, 5], which trigger their import into cellular endosomes. Within the cell, hypomethylated DNA interacts with TLR9 and triggers a response through the NF-κβ signaling cascade [6, 7]. The immunostimulatory response to TLR9 signaling in immune cells can modulate the course of diseases, including autoimmune diseases [8], infectious diseases [9], cancers [10], and allergies [11]. This immunostimulatory effect has also been exploited as a vaccine adjuvant [12, 13].

Investigation of TLR9 signal induction in different immune cell types, such as plasmacytoid dendritic cells (pDCs) and B cells, has led to the development of active synthetic CpG ODNs. These ODN sequences incorporate various patterns of phosphorothioate (PS) linkages in the backbone of the single-stranded DNA (ssDNA) [14], as PS-containing ODNs resist enzymatic degradation for improved stability. Different classes of CpG ODNs (referred to as CpG-A, CpG-B, and CpG-C) induce differential immune responses in pDCs and B cells [15]. CpG-A induces high levels of IFNα and IFNβ in pDCs but little B cell activation. Conversely, CpG-B is a direct and potent activator of B cells, and promotes maturation of dendritic cells, but stimulates the production of IFNα and IFNβ only weakly. CpG-C shares the inducing effects of CpG-A and CpG-B, inducing both active IFNα secretion and robust B cell activation.

CpG ODNs, in particular the class B ODN CpG 7909, have been used as vaccine adjuvants to enhance vaccine-specific antibody production and function in mice and humans [16–18]. However, the concentration of CpG-B (PS) ODNs used in humans induces unwanted reactogenicity, such as injection site soreness and fever. Such reactogenicity may derive in part from CpG-B-induced cytokine production [19, 20]. Nonetheless, in contrast to CpG-A or CpG-C, CpG-B may serve as a more effective vaccine adjuvant by enhancing vaccine-specific antibody responses with potentially more limited pro-inflammatory cytokine production. Agents that synergize with CpG-B-mediated induction of B cell antibody responses hold promise to reduce vaccine reactogenicity in patients by limiting the CpG-B dose required. Of note, mitochondrial transcription factor A (TFAM) was shown to synergistically enhance CpG-A-mediated IFNα production from human pDCs [21, 22], but the effect of TFAM on CpG-B and -C -mediated antibody response is unknown. TFAM has been shown to bind DNA with nanomolar affinity [23–25], and could potentially shed light on novel treatments for autoimmune diseases such as lupus where self DNA creates unwanted activation of TLR9 receptors [8, 26].

TFAM is a member of the high mobility group box protein (HMGB) family of DNA binding proteins [27, 28] and is similar in structure to HMGB1 [27–29]. HMGB1 is a both a chromosomal protein residing in the nucleus, and a damage-associated molecular pattern (DAMP) protein present extracellularly, where it signals through TLR2, TLR4, and RAGE [30–32] (reviewed in [27, 28, 33]). Furthermore, HMGB1 has been shown to alter CpG-mediated TLR9 activation [34]. TFAM functions both as a transcription factor and as a nucleoid protein for the mitochondrial genome [27, 33, 35]. It has high affinity for both double-stranded DNA (dsDNA) [23, 25] and modified dsDNA [36, 37], and is abundant in the mitochondrial fraction of necrotic cells [3, 22]. Recently TFAM has also been found to act as a DAMP in brain microglia and hemorrhagic shock [38, 39], and thus might also, like HMGB1, have an immune-modulating function.

To determine whether TFAM modulates TLR9-mediated antibody production in human B cells, we conducted complementary molecular and cellular studies focused on the immunostimulatory humoral effects of CpG-B and -C (PS). Using fluorescence anisotropy (FA), we found that TFAM binds to CpG-A, -B, and -C directly with high affinity. TFAM also attenuates CpG-B and -C (PS) binding and uptake by B cells and modulates TLR9-dependent CpG-induced antibody production. These findings suggest an alternative role for TFAM in CpG- mediated antibody responses compared to pDC IFNα production. Although these findings may preclude the use of TFAM as a CpG-B enhancing vaccine adjuvant, TFAM may be useful for decreasing the pathogenic consequences of exposure to hypomethylated DNA in the context of trauma, autoimmune disease, or infection [8, 9, 26, 40, 41].

## Materials and Methods

### DNA purification

**Fig 1:**
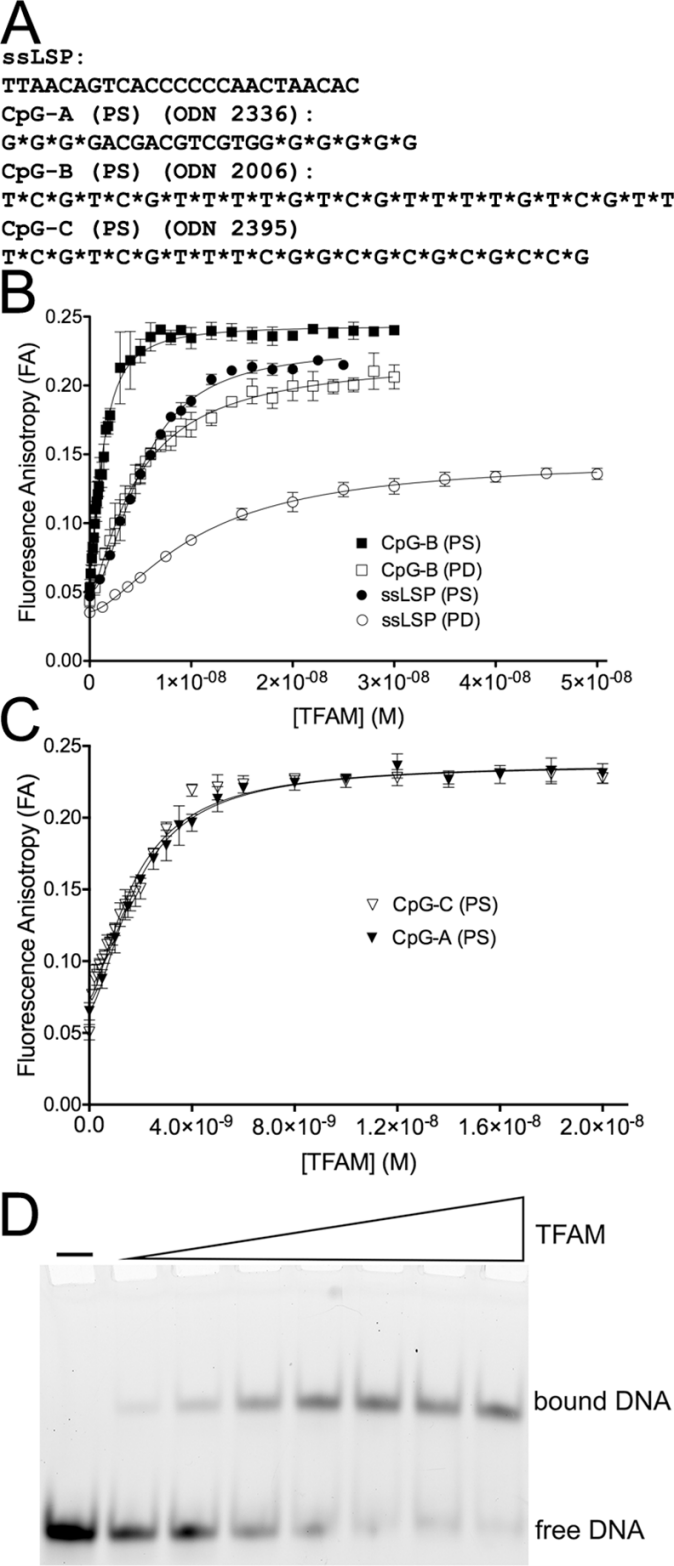
TFAM binds CpG (PS) ODNs with nanomolar affinity. **(A)** DNA sequences used for fluorescence anisotropy spectroscopy. Sequences of single strands are shown in the 5’ to 3’ direction, and an asterisk (*) indicates a phosphorothioate (PS) linkage. The template strand of the human mitochondrial lights strand promoter is designated ssLSP, and other CpG sequences have been described [14, 42]. **(B)** TFAM binding isotherms, obtained from FA data, of FM-labeled CpG-B ODN and ssLSP ODN with phosphodiester (PD) or phosphorothioate (PS) backbones (n = 4). **(C)** TFAM binding isotherms of FM-labeled CpG-A (PS) and CpG-C (PS) obtained from FA data. **(D)** EMSA of TFAM binding to FM-labeled phosphorothioate CpG-B (PS) ODN. The concentration of FM-CpG-B (PS) was 5 nM, and the TFAM concentration was varied from left to right: 0 nM, 1 nM, 2 nM, 3 nM, 4 nM, 5 nM, 6 nM, and 8 nM, respectively.

The DNA used in this study in both phosphodiester (PD) and phosphorothioate (PS) forms, as well as fluorescein 5’-6FAM (abbreviated as FM)–labeled DNA, was produced synthetically (Integrated DNA Technologies, Coralville, IA) [14] (Fig 1A). All ODNs were purified through chromatographic separation on a 5 ml DEAE column with a gradient of NaCl from 0 to 2.5 M for the PS ODNs and 0 to 1 M for PD ODNs.

### TFAM expression and purification

TFAM was expressed and purified from *E*. *coli* as previously described [23]. TFAM was then exchanged into buffer (50 mM HEPES-Na, pH 7.4, 150 mM NaCl, 1 mM DTT). This method yields highly pure TFAM (S1 Fig).

### Fluorescence anisotropy (FA) spectroscopy

In order to measure the TFAM binding affinity for the different types of DNA, fluorescence anisotropy experiments were conducted on a Jobin Yvon Horiba Fluorolog 3 fluorescence spectrometer. TFAM was titrated into 500 pM of various FM-labeled DNA fragments in a 1 ml cuvette containing 50 mM HEPES-Na, pH 7.4, 150 mM NaCl, 1 mM DTT in a cuvette thermostatted at 20°C. For measuring FA, spectra were recorded in the polarization mode with an excitation wavelength of 490 nm and a 5 nm bandpass. Emission was measured at 520 nm with a bandpass of 9 nm. The dissociation constants (K_D_) were obtained by fitting the data to the Hill equation using data from a minimum of three independent experiments.

### Circular dichroism (CD) spectroscopy

In order to determine the structural identity of DNA fragments used in this study (double [ds] or single strand [ss]), circular dichroism experiments were performed on a Jasco J-815 CD spectrometer equipped with Lauda Brinkman ecoline RE106 temperature controller. 20 μM DNA in 10 mM Na-phosphate buffer, pH 7.4, and 150 mM NaCl was examined at a temperature of 20°C in a cuvette with a path length of 1 mm. Circular dichroism was measured in millidegrees from 195 nm to 300 nm with a bandpass of 1 nm and a step size of 0.2 nm. Six scans of each sample were averaged. All spectra have had the buffer background subtracted.

### Electrophoretic mobility shift assay (EMSA)

The formation of discrete TFAM-DNA complexes was monitored using EMSA. FM-labeled CpG-B (PS) ODN at a concentration of 5 nM was titrated with increasing concentrations of TFAM prior to nondenaturing PAGE (8% acrylamide with a ratio of 37.5:1 acrylamide:N,N′-methylene-bis-acrylamide; BioRad, Hercules, CA). The gel and electrophoresis buffer was 0.33 x TBE (90 mM Tris, 90 mM boric acid, 2 mM EDTA). The gels were prerun at 125 V for 30 min prior to sample loading and then electrophoresed at 75 V for 1 h. Gels were imaged using a Storm 860 Scanner (Molecular Biosystems, San Diego, CA) with the excitation laser set to 488 nm and the emission cutoff at 520 nm.

### Cell isolation and stimulation

Peripheral blood mononuclear cells (PBMCs) were isolated from heparinized blood of healthy adult donors using standard ficoll density gradient separation. Cells were frozen and stored in liquid nitrogen until use. PBMCs (2x10^5^ cells/well) were cultured in RPMI with 10% heat-inactivated fetal bovine serum and 10 μg/ml gentamicin in 96 well flat-bottomed plates with CpG class B (Integrated DNA Technologies) (referred to here as CpG-B (PS)) with or without TFAM at the indicated concentrations. For blocking experiments, we used colchicine (150 ng/ml; Sigma, St. Louis, MO), an inhibitor of microtubule-mediated uptake, and ODN TTAGGG (“iODN”; 10:1 ratio of iODN to CpG-B (PS), Invivogen, San Diego, CA), an inhibitory oligonucleotide and a TLR9 antagonist. After 7 days, culture supernatants were tested for IgG antibody production by enzyme-linked immunosorbent assay (ELISA) as previously described [43]. The Colorado Multiple Institutional Review Board (COMIRB) specifically approved this study and the use of all samples in these studies (#050993). Participants provided their written informed consent to participate in this study.

### CpG binding by B cells

PBMCs (1x10^6^ cells) were incubated with media, 0.08 μM FM-labeled CpG-B (PS) and varying doses of TFAM for 1 h; and then washed twice and labeled with Pacific blue-conjugated mouse anti-human CD19 (Biolegend, San Diego, CA). Washed cells were fixed in 2% paraformaldehyde and analyzed both by flow cytometry. Flow cytometry acquisition was performed on a LSR II (BD Bioscience, San Jose, CA) and analyzed using FlowJo software (Treestar, Ashland, OR).

### Statistical analysis

Data were analyzed with GraphPad Prism Version 6 for Mac OS X (La Jolla, CA) using Freidman test with Dunn’s multiple comparison post-test or Wilcoxon signed rank test to compare antibody production (for paired, non-parametric data) and RM one-way ANOVA with Holm-Sidak’s multiple comparison post-test or paired t-test for flow cytometry data analyses (for paired, parametric data). P-values of less than 0.05 were considered as statistically significant.

## Results

### TFAM binds directly to the CpG-A, B, and C phosphorothioate ODNs with high affinity

In its role as a transcription factor and nucleoid protein, TFAM binds to double stranded DNA with high affinity in a manner that is selective for mitochondrial DNA (mtDNA) promoters, as well as non-sequence specifically throughout the mtDNA [23, 25]. As previous studies have shown that TFAM and CpG-A DNA cooperate to increase INFα production in pDCs through a TLR9-mediated mechanism [21, 22], we investigated whether the mechanism could involve direct binding of TFAM to CpG ODNs. CpG-A and the closely related CpG-B and C ODNs have sequences and structures that are distinct from TFAM binding sites, and one of the CpGs has been shown recently to be single stranded [44]. Therefore, we first measured the binding affinity of purified TFAM for the synthetic CpG-B ODN (Fig 1A) using fluorescence anisotropy (FA). FA is a measure of the effective tumbling rate of a fluorophore in solution; this rate generally slows and FA increases with ligand binding. As TFAM was titrated into FM-labeled CpG-B (PS) ODN, the FA increased (Fig 1B). The formation of a larger complex indicates that TFAM binds directly to the fluorophore–labeled DNA. The dissociation constant (K_D_) value for the TFAM-CpG-B (PS) ODN complex was 1.30 nM ± 0.03 (Table 1). Thus, TFAM binds directly to CpG-B (PS) with a high affinity, and this interaction is nearly fourfold tighter than TFAM binding to one of its natural targets, which is the double-stranded mitochondrial light strand promoter DNA (K_D_ = 4.4 ± 0.3 nM) [23, 24]. The similarly high affinity of TFAM for dsDNA and CpG (ssDNA) was unusual and unexpected.

**Table 1:** Protein/DNA dissociation constants K_D_(M).

	TFAM	TFAM Box A	TFAM Box B
**CpG-A (PS)**	1.83 ± 0.07 x10^-9^	7.12 ± 3.8 x10^-8^	2.31 ± 0.30 x10^-7^
**CpG-B (PS)**	1.30 ± 0.03 x10^-9^	1.04 ± 0.10 x10^-8^	not detectable
**CpG-C (PS)**	1.77 ± 0.08 x10^-9^	7.01 ± 0.07 x10^-9^	not detectable
**CpG-B (PD)**	4.66 ± 0.20 x10^-9^		
**ssLSP (PS)**	5.06 ± 0.10 x10^-9^		
**ssLSP (PD)**	1.02 ± 0.05 x10^-8^		

We considered the potential mechanisms underlying the high affinity of TFAM for the CpG-B (PS) ODN. To test for an effect of the DNA sequence, we compared the binding affinity of TFAM for CpG-B (PS) to the single-stranded mitochondrial light strand promoter DNA that had been synthesized with a phosphorothioate rather than a native phosphodiester backbone (ssLSP (PS) Fig 1A). The binding affinity of TFAM for FM-ssLSP (PS) (Fig 1B) was nearly 4 fold weaker (K_D_ = 5.06 ± 0.10 nM) than for the FM-CpG-B (PS) ODN (Table 1). This result indicates that TFAM binds preferentially to the CpG-B (PS) ODN sequence relative to a non-CpG single stranded phosphorothioate-containing ODN (Fig 1B) [23, 24].

We next examined the effect of the phosphorothioate (PS) versus the phosphodiester (PD) backbone linkages on TFAM binding to two otherwise identical sequences. Using FA, we found that TFAM bound more tightly to the CpG-B (PS) ODN than to the CpG-B (PD) ODN (compare K_D_ of 1.30 ± 0.03 nM to K_D_ of 4.66 ± 0.20 nM, respectively) (Fig 1B and Table 1). Similarly for the PS and PD versions of the non-CpG sequence, ssLSP DNA, TFAM preferred binding to the PS version compared with the PD version (K_D_ of 5.06 ± 0.10 nM versus 10.2 ± 0.5 nM, respectively) (Table 1). Therefore, the presence of the phosphorothioate backbone increases the binding affinity of TFAM for both CpG and ssLSP ODNs by a factor of two to three over the natural phosphodiester backbone. Taken together, these results indicate that both sequence and backbone composition contribute to the tight binding of TFAM to ODNs such as CpG-B (PS).

The high affinity of TFAM for the CpG-B ODN could be specific to this CpG class of ODNs or also could extend to other classes of CpG (PS) ODNs. Therefore, we tested how tightly TFAM bound to representatives of two structurally distinct CpG classes, CpG-A (PS) and CpG-C (PS) (Fig 1A). Using FA, as TFAM was titrated into either FM-CpG-A (PS) or FM-CpG-C (PS), the FA increased indicating direct binding of TFAM to the ODNs (Fig 1C). The binding affinities of TFAM for CpG-A (PS) and CpG-C (PS) were nearly identical to each other (K_D_ values of 1.83 ± 0.07 nM and 1.77 ± 0.08 nM, respectively) and to the CpG-B (PS) ODN (Table 1). Therefore, TFAM is not specific for CpG-B (PS), but can bind similarly tightly to all of the CpG classes tested here. Remarkably, all three classes of CpG (PS) ODNs bind to TFAM ~2–3 fold more tightly than TFAM binds to any other ODN tested, including the (PS) and (PD) forms of its naturally occurring single stranded or double stranded cognate binding site in the light strand promoter of mitochondrial DNA [23, 24].

TFAM has the unique ability to bind DNA both sequence-specifically and non-sequence specifically. It binds with similar affinity to both types of sequences and forms stable, discrete and ordered complexes [23–25, 29, 45]. In order to determine whether the interaction between TFAM and the CpG-B (PS) ODN also leads to formation of a stable and discrete complex, we examined the complexes formed with CpG-B (PS) ODNs using EMSA. Unstable protein-DNA complexes or highly fluctuating structures would be expected to appear as a smear in the gel. However, TFAM binds to CpG-B (PS) in a dose-dependent manner with a single well-defined shift in the position of the DNA within the gel (Fig 1D). This result indicates that TFAM does indeed form a complex with the CpG-B (PS) ODN that is distinct and stable to the forces of electrophoresis.

**Fig 2:**
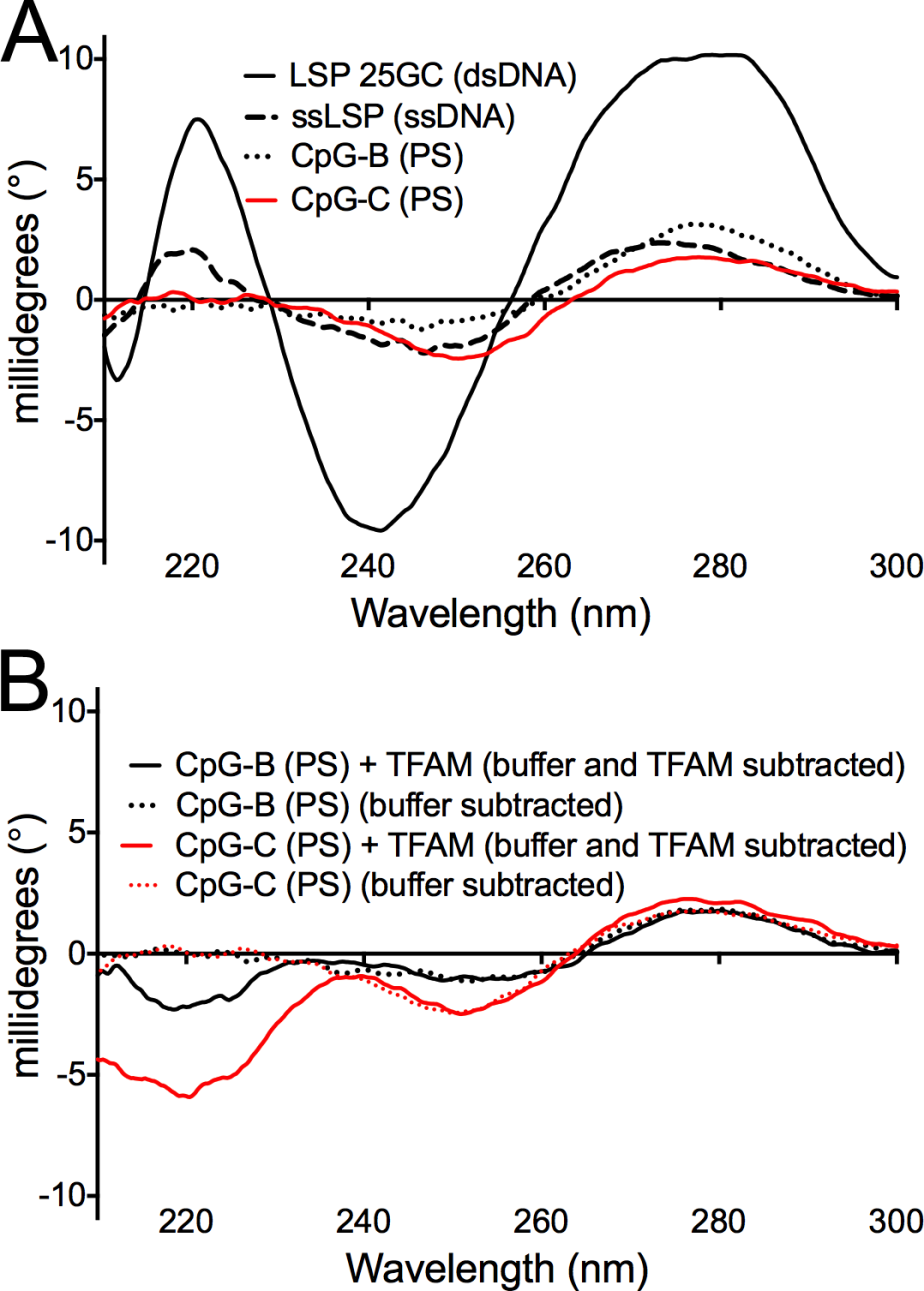
**TFAM does not alter the secondary structure of CpG-B or CpG-C (A)** Circular dichroism (CD) analysis of CpG-B (PS) and CpG-C ODNs. CD spectra measured in millidegrees are shown for the 25mer dsLSP DNA (solid line), ssLSP ODN (dashed line), CpG-B (PS) ODN (dotted line), and CpG-C (PS) (red line) at a concentration of 20 μM. **(B)** CD analysis of CpG-B (PS), and CpG-C (PS) in the presence and absence of TFAM. The DNA concentration was 10 μM for both samples. In the CpG-B (PS) and CpG-C (PS) spectra, buffer was subtracted, whereas for the CpG-B (PS)+TFAM and CpG-C (PS)+TFAM spectra, the CD contribution both from the buffer and TFAM was subtracted. All CD experiments were conducted at 20°C in 10 mM Na-phosphate buffer and 150 mM NaCl.

A third possible contribution to the high affinity of TFAM for the CpG-B (PS) ODN is that TFAM selectively binds to a double-stranded and not the single-stranded form of the ODN. Previous studies have already shown that CpG-A can form higher order G-quadruplex structures [46, 47]. Although a recent structural analysis of the TLR9-CpG-A-like ODN complex revealed that this DNA adopts a single stranded form [44]. We therefore examined the secondary structure of the CpG-B (PS) and CpG-C (PS) ODN and control ODNs in the absence and presence of TFAM to determine if CpG-B (PS) and CpG-C (PS) could form higher order structures. Circular dichroism spectroscopy (CD), which uses circularly polarized light to characterize the secondary structure of DNA, gives distinctly different CD spectra for ssDNA and dsDNA [48, 49]. The CD spectra of CpG-B (PS), CpG-C (PS), and ssLSP are similar and lack the characteristic maximum and minimum seen for dsLSP DNA (Fig 2A). Thus, the CpG-B (PS) and CpG-C ODNs exist in a predominantly single-stranded form under the conditions of this experiment in the absence of TFAM. These CD data for CpG-B (PS) are in agreement with previous atomic force microscopy studies [47].

Finally, to rule out the possibility that TFAM promotes the formation of a double-stranded form of the CpG-B (PS) ODN as a means to achieve a higher affinity, we conducted CD experiments with CpG-B (PS) in the absence and presence of TFAM (Fig 2B). After the subtraction of appropriate buffer and TFAM spectra from the CpG-B (PS) and CpG-C (PS) alone spectra, and from the CpG-B (PS) and CpG-C (PS) plus TFAM CD spectra, the spectral signal of the remaining DNA for each sample was relatively consistent and similar to the ssDNA alone spectra. Therefore, binding of TFAM does not promote any double-stranded character in the CpG-B (PS) or CpG-C ODNs. Together, these data support the model that TFAM selectively recognizes both the sequence and backbone composition of CpG ODNs as a single complex comprising TFAM and the single-stranded form of the CpG-B and CpG-C ODN.

### CpG-B (PS)-mediated IgG production by human B cells is altered by TFAM and is dependent on CpG concentration

**Fig 3:**
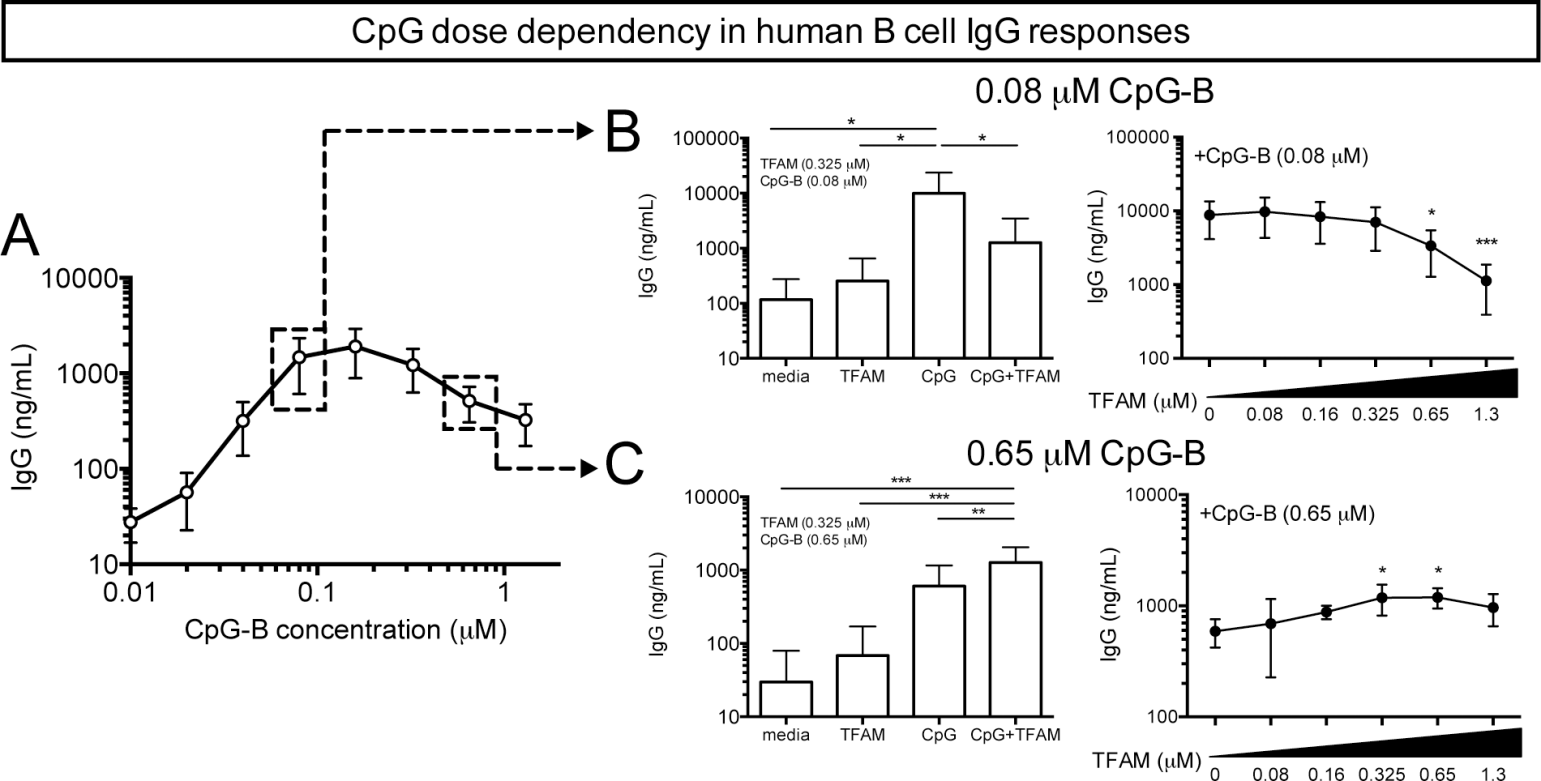
TFAM alters CpG (PS)-mediated IgG production from human B cells. **(A)** IgG production by peripheral blood mononuclear cells (PBMCs; n = 5) stimulated with varying doses of CpG-B (PS) for 7 days. Mean±S.E.M. **(B)** PBMCs (n = 7) stimulated with a low dose of CpG-B (PS) (0.08 μM) with or without 0.325 μM TFAM (left panel) or increasing concentrations of TFAM, as indicated (right panel). Mean+S.D. [Note: 2 of the 7 donors had higher IgG expression at baseline and with stimulation than the others. Without these two subjects, the scales on the all the 0.08 uM IgG charts will be similar]. **(C)** PBMCs (n = 3–7) stimulated with a high dose of CpG-B (PS) (0.65 μM) with or without 0.325 μM TFAM (left panel) or increasing TFAM concentrations, as indicated (right panel). Mean+S.D. * P<0.05, ** P<0.01, *** P<0.001.

CpG ODNs can induce IFNα production from pDCs (CpG-A), antibody production from B cells (CpG-B) or both (CpG-C) [15]. Although TFAM may enhance CpG-A-induced IFNα production from human pDCs [21], the effect of TFAM on CpG-B- and -C-mediated antibody production from B cells had not been characterized. Thus, we stimulated peripheral blood mononuclear cells (PBMCs), which contain B cells, T cells, monocytes and a small number (<1%) of plasmacytoid dendritic cells (pDC’s), with CpG-B (PS) in the presence or absence of TFAM and measured production of immunoglobulin G (IgG) antibody. These initial studies with the B cell-specific CpG-B (PS) showed that CpG-B (PS) alone induced IgG production from human B cells in an ascending-descending dose-dependent manner, but with an attenuation of antibody production at the higher CpG doses (Fig 3A).

From this dose response, we selected a low dose (0.08 μM [0.625 μg/ml]) (Fig 3B) and a higher dose (0.65 μM [5 μg/ml]) of CpG-B (PS) (Fig 3C) for TFAM co-incubation. The high dose of CpG-B (PS) is similar to CpG concentrations used in many *in vitro* studies [50, 51] including those for IFNα production (with CpG-A) [20]. As per the initial dose response curve (Fig 3A), when stimulating with CpG-B alone, a relatively lower dose of CpG-B (0.08 μM) elicited more IgG production than did the higher dose (3668 vs 414 ng/ml median values, respectively). As we are using recombinant TFAM, to exclude LPS contamination as contributor to our results, PBMCs were also stimulated with LPS, TFAM buffer or an arbitrary protein isolated in the same manner as TFAM. None of these conditions induced antibody production, but it is known that LPS alone does not normally stimulate human B cells [38, 52]. Moreover, CpG-induced IgG production from PBMCs was not affected by addition of the arbitrary protein used at a same concentration as TFAM (data not shown). TFAM alone had no appreciable effect on IgG production (Fig 3B and 3C). The addition of TFAM to the lower dose of CpG-B (PS) resulted in a dose-dependent diminution, rather than enhancement, of IgG production (Fig 3B). At this lower CpG dose (a TFAM:CpG-B (PS) ODN molar ratio of 8:1) TFAM attenuated IgG production to levels similar to the media alone control. Conversely, with the higher dose of CpG-B (PS) (0.65 μM), the addition of TFAM significantly enhanced IgG production (Fig 3C). This enhancement was dependent on the concentration of TFAM present, with the highest IgG production seen at a TFAM:CpG-B molar ratio of 1:1. Thus, in the presence of high doses of CpG-B (PS), TFAM shifts the CpG dose response curve (as observed in Fig 3A) to the left (increased IgG). At lower CpG-B (PS) concentrations, the binding of TFAM to CpG also shifts the curve to the left, but, in this case, down the CpG dose response curve (decreased levels of IgG). Thus, TFAM may both enhance and reduce IgG production by human B cell stimulated with CpG, depending on both the dose of CpG (PS) used as well as the ratio of TFAM to CpG.

### TFAM inhibits IgG production induced by both CpG-B and -C (PS) by preventing CpG binding to B cells

**Fig 4:**
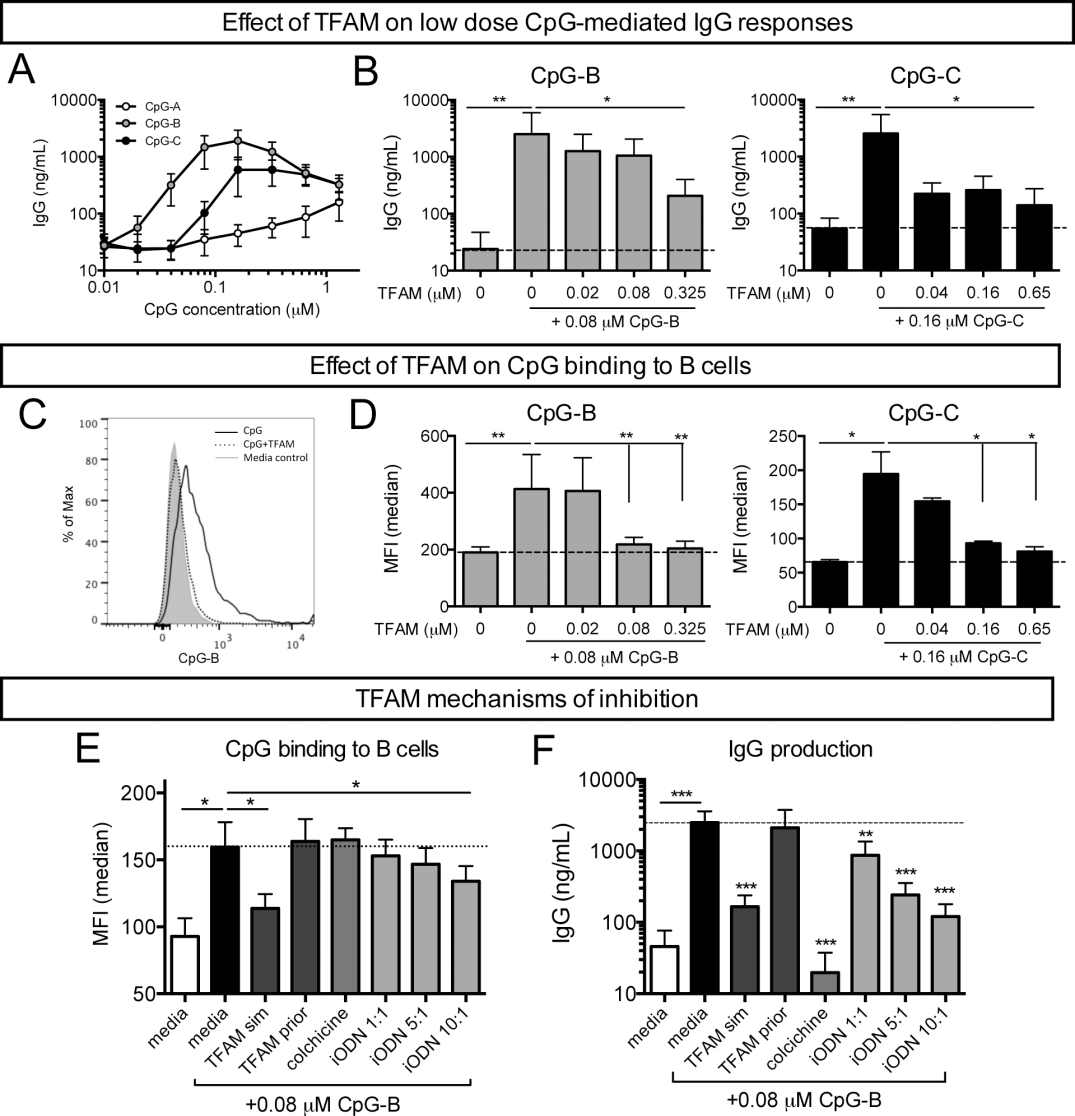
TFAM attenuates antibody production by preventing CpG binding to B cells. **(A)** IgG production by PBMCs (n = 3–5) stimulated with varying doses of CpG-A, -B or -C (PS) for 7 days. Mean±S.E.M. **(B)** IgG production from PBMCs (n = 4) stimulated with CpG-B or -C with increasing concentrations of TFAM. Mean+S.D. **(C)** Representative flow cytometric histogram of FM-labeled CpG-B staining on CD19^+^ B cells in PBMCs after one hour incubation with CpG-B alone (0.08 μM, black line), CpG-B (0.08 μM) + TFAM (0.325 μM, dotted line) or media control (gray filled). **(D)** Median fluorescence intensity (MFI) of FM-labeled-CpG-B (gray bars) or–C (black bars) staining on B cells in PBMCs (n = 7) after one hour of incubation with increasing concentrations of TFAM. P-values are compared to CpG only control. **(E)** MFI on B cells and **(F)** IgG production after PBMCs (n = 4) were stimulated with TFAM at the same time as CpG-B (TFAM sim; 0.325 μM) or prior to CpG-B addition (TFAM prior; 0.325 μM), colchicine (blocks CpG endocytosis; 150 ng/ml) or a TLR9 antagonist (iODN, blocks TLR9 signaling by outcompeting CpG for binding; molar ratios of iODN to CpG-B). Mean+S.D. * P<0.05, ** P<0.01, *** P<0.001.

We were interested in whether the effect of TFAM on B cell IgG production was specific to CpG-B (PS) or universal to all CpG classes. Thus, we initially stimulated PBMCs and performed a dose curve with CpG-A, -B and -C (PS) to determine the effect of IgG production utilizing each CpG class. Both CpG-B and -C (PS) induced robust dose-dependent levels of IgG, although CpG-B (PS) induced higher levels than CpG-C (PS) (Fig 4A). Responses to CpG-A (PS) were limited so this class was not tested further. Based on the dose response curves, we stimulated PBMCs with a low dose of CpG-B and -C (PS) (0.08 μM and 0.16 μM, respectively) in the presence or absence of increasing molar ratios of TFAM. A higher concentration of CpG-C (PS) was used based on the lower levels of IgG production observed in the initial dose titrations. Consistent with our earlier observations, IgG production from both CpG-B- and -C (PS)-stimulated PBMCs was attenuated with the addition of increasing concentrations of TFAM (Fig 4B). Thus, similar to the ability of TFAM to bind both CpG-B and -C (PS), TFAM also inhibits IgG production induced by both classes of CpG. These data suggest that TFAM may alter the free concentration of CpG (PS) available to stimulate B cells potentially by sequestering the CpG in a TFAM-CpG (PS) complex.

Thus, we next considered whether the effects of TFAM on CpG (PS)-mediated IgG production was due to TFAM preventing CpG binding to B cells. As detected by flow cytometric analyses, the addition of TFAM decreased the amount of fluorescent FM-labeled CpG ODNs bound to B cells (curve shift to the left) compared with CpG-B (or C) (PS) alone in a TFAM dose-dependent manner (Fig 4C and 4D). Consistent with the effects on antibody production, the reduction of CpG-B (PS) binding was greatest, and most similar to media alone control, at a TFAM:CpG-B (PS) molar ratio of 1:1 or above (4:1). Moreover, TFAM also inhibited B cell binding of CpG-B (PD) that has a phosphodiester backbone (S2 Fig), suggesting that TFAM influences the cellular binding of CpG ODNs independent of the presence of the physiologically relevant phosphodiester or constructed phosphorothioate backbones.

We additionally tested whether the mechanism of inhibition by TFAM was to prevent CpG-B (PS) from binding to B cells or to block the receptors that mediate uptake of CpG-B (PS) into cells. We exposed B cells to TFAM concurrently with (“TFAM sim”) or prior to (“TFAM prior”) CpG-B (PS) stimulation and compared levels of B cell binding to those found with colchicine (prevents CpG endocytosis) and a competitive inhibitor (iODN) of TLR9 (the intracellular receptor for CpG-B). TFAM added to cells prior to stimulation and washing did not prevent CpG-B (PS) B cell binding (Fig 4E) or IgG production (Fig 4F). In contrast, TFAM added simultaneously with CpG-B (PS) resulted in decreased binding of CpG-B (PS) to B cells. Moreover, TFAM and CpG-B (PS) added simultaneously exhibited similar reductions in B cell surface binding and antibody production to that of iODN, a direct competitive inhibitor of CpG for TLR9 binding. However, unlike TFAM, colchicine, which is an indirect inhibitor of TLR9 signaling that does not interact with CpG-B (PS) directly, did not block CpG-B (PS) binding, but IgG production was still reduced. Thus, TFAM directly binds to CpG ODNs, inhibits CpG binding to B cell surface receptors and thereby attenuates CpG-mediated antibody production by B cells. We next evaluated the components of the TFAM protein that bound directly to CpG to account for this inhibition.

### TFAM Box A binds to CpG-A, -B and -C, but is insufficient to inhibit CpG- mediated antibody production

**Fig 5:**
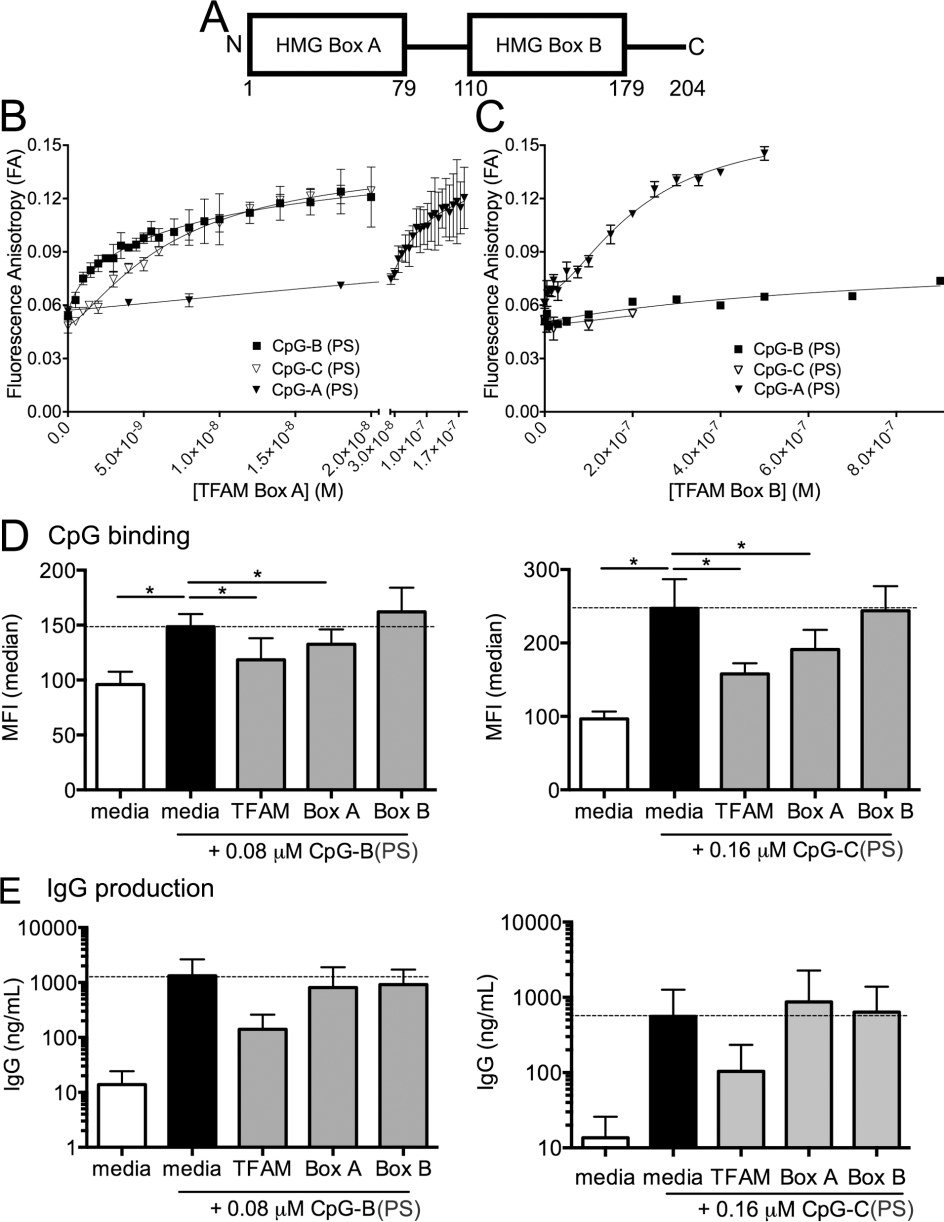
TFAM Box A reduces CpG-B and -C binding to B cells but does not inhibit IgG production. **(A)** Schematic diagram of TFAM functional regions, with amino acids numbered. FA binding isotherms obtained for FM-labeled CpG-B (PS) and CpG-A (PS) binding to **(B)** TFAM high mobility group (HMG) Box A and **(C)** Box B. CpG-B or -C (PS) binding **(D)** and IgG production **(E)** of PBMCs (n = 4) stimulated with CpG-B or–C (PS) with or without full-length TFAM (0.325 μM) or the individual DNA binding domains (Box A (0.325 μM) or Box B (0.325 μM)). Dotted line indicates level of stimulation with CpG alone. * P<0.05.

TFAM is a 204 amino acid long protein composed of two HMG box domains separated by a linker and C-terminal tail [24] (Fig 5A). Previous work from our lab [23] and others [25] has shown that TFAM Box A can bind DNA, whereas Box B alone binds very weakly. However, either Box A or Box B with CpG-A DNA increase TNFα production from pDCs compared CpG-A alone [22]. Our findings that TFAM binds directly to CpG-A, -B, and -C raised the possibility that the individual HMG boxes might also bind CpG ODNs. To measure the binding affinity of Box A and Box B to the CpG-A (PS), CpG-B (PS), and CpG-C (PS) ODNs, we again employed FA. We found that Box A alone bound FM-CpG-B (PS) and FM-CpG-C (PS) with similar K_D_ values, of 10.4 nM ± 1.0, and 7.01 ± 0.07 nM, respectively (Fig 5B), approximately an order of magnitude weaker than full-length TFAM (Fig 1B and 1C). On the other hand, Box A bound CpG-A (PS) with a K_D_ of 71.2 ± 38 nM, which is approximately an order of magnitude weaker binding than for the CpG-B (PS) and CpG-C (PS) ODNs, and nearly two orders of magnitude weaker binding than full length TFAM binding to CpG-A (PS) ODN (Fig 1C). In contrast, Box B alone bound CpG-B (PS) and CpG-C (PS) so weakly that an accurate K_D_ could not be measured (Fig 5C), whereas surprisingly Box B bound CpG-A with a K_D_ of 231 ± 30 nM. These data suggest that each constituent of TFAM contributes to and is required for high binding affinities in the 1 nM range of full-length TFAM to CpG (PS) ODNs. Box A can bind CpG-B (PS) and CpG-C (PS) with moderate affinity in the ~10 nM range, while binding CpG-A (PS) with weak affinity in the ~100 nM range. Conversely, Box B cannot bind CpG-B or -C (PS), but can bind CpG-A (PS) weakly in the ~100 nM range.

After determining that TFAM Box A, but not Box B binds to CpG-B (PS), we next investigated the ability of the individual HMG box domains to modulate CpG-B (PS)-mediated IgG production. Although Box A did result in a modest reduction in CpG-B (PS) binding to B cells (Fig 5D), this reduction did not impair IgG production (Fig 5E). Moreover, consistent with the binding results, Box B alone neither inhibited CpG-B (PS) binding to B cells nor prevented IgG production. Thus, both DNA binding domains of TFAM are required for inhibition of CpG-B (PS)-mediated antibody production by B cells.

## Discussion

We demonstrate that TFAM binds directly and with high affinity to three classes of CpG oligonucleotides (ODN), CpG-A, -B, and -C (PS), *in vitro*. This interaction limits binding of CpG-B and -C to B cells and modulates CpG-mediated antibody production from human B cells. We further demonstrate the importance of the CpG dose as a determinant of the magnitude of antibody production and the effects of TFAM. At lower doses of CpG, TFAM inhibits IgG production, whereas at higher CpG doses, TFAM enhances antibody levels. These potentially disparate effects are reconciled by the ability of TFAM binding to shift the concentration of free CpG available in solution to bind B cells to elicit the dose-dependent antibody responses along the curve. This study highlights the importance of the CpG dose and the interplay between TFAM and CpG. Furthermore, these intriguing results provide the impetus for future studies to better dissect the dose response and TFAM effects for other CpG classes, as well as for studying different cellular responses to optimize the use of this TLR9 agonist and adjuvant to enhance immune responses to vaccines *in vivo*.

The remarkably high affinity of TFAM for the CpG-B (PS) ODN, on par with the affinity of TFAM for its natural binding site, was quite unexpected. As a nucleoid protein, TFAM has the ability to recognize different dsDNA sequences [23–25, 35]. However, this ability usually comes at the price of overall affinity; for example, the chromosomal proteins HMGB1 [53] and Sac7d [54] recognize DNA in a non-sequence-specific manner with lower affinities typically in the micromolar, not the nanomolar range described herein. Unlike TFAM [25], these proteins also generally have even weaker affinities for ssDNA. This is the first study to characterize binding of TFAM with the CpG-B ODN, revealing that TFAM has an unexpected selectivity for the phosphorothioate backbone in the CpG-A, -B, and -C (PS). Few other proteins have been shown to recognize this feature of DNA with such high affinity. The Ff phage gene 5 protein binds to ssDNA with an affinity that is considerably weaker overall compared to TFAM, but the inclusion of phosphorothioate linkages in the DNA backbone dramatically increased the affinity for PS-DNA relative to PD-DNA [55, 56]. We are pursuing the molecular basis for the effect of the phosphorothioate linkage on TFAM recognition of ODNs.

The recognition and binding of CpG ODNs by TFAM, in turn, can alter CpG-mediated antibody production by human B cells. The ability of TFAM to enhance antibody production by higher dose CpG (CpG-B, -C) is consistent with the previously reported enhancement of CpG-A-mediated cytokine production (IFNα) from pDCs using a single, high dose of CpG-A (PS) (0.5 μM [~3.4 μg/ml]) [21]. Furthermore, our observations that both TFAM Box A and Box B have the ability to bind CpG-A ODN supports previous findings that both of these regions of TFAM can alter CpG-A mediated IFNα production from pDCs [22]. However, we also found that at lower concentrations of CpG-B and -C, TFAM inhibited antibody production by B cells. An explanation for the difference between these findings for the CpG-A compared with CpG-B and CpG-C ODNs is that different responses to TFAM may occur in the context of different cell types (B cells versus pDCs) and their requirements for TLR9 activation (early endosome for CpG-A versus late endosome for CpG-B) [57]. Whether TFAM could also inhibit IFNα production by pDCs at lower CpG-A doses, by binding free CpG and limiting binding to pDC, has not been determined.

Another striking discovery from this study was the ability of TFAM Box B to bind CpG-A (PS) with a modest affinity. Previous studies have shown that TFAM box B lacks the ability to bind specific dsLSP DNA [23] as well as non-specific dsDNA and ssDNA [25], whereas TFAM Box A was found to bind DNA in these studies. Alternatively, the interaction of TFAM Box B with ds- and ss-DNA, within the context of full-length TFAM, could be greatly enhanced by interactions that occur with Box A or the linker between the boxes [23]. Thus, the unique structure formed by CpG-A DNA [46, 47] can allow for weak binding of both TFAM Box A and Box B.

Finally, hypo- or unmethylated DNA, such as in CpG ODNs, is a pathogen- or damage-associated molecular pattern that elicits cellular responses, including neutrophil activation [3] and IFNα and antibody production via the engagement of TLR9. Given the unique ability of TFAM to bind all three classes of CpG (PS) ODN with such high affinity, TFAM may play complementary roles in augmenting or inhibiting CpG-mediated immune responses depending on the concentration and target cell. Indeed, this study demonstrates that TFAM can inhibit CpG-mediated antibody production by binding to free CpG ODN and preventing direct CpG cellular interactions with human B cells. As TFAM can bind so tightly to generic ssDNA, TFAM could potentially also be used to suppress aberrant DNA-mediated immune activation and autoantibody production. Moreover, these studies *in vitro* provide a conceptual basis to characterize the modulatory interactions of hypomethylated DNA and its varied physiologic ligands, such as TFAM, and receptors in such diverse and pathologic conditions as traumatic injury [40, 41], sepsis and potentially in fetal DNA-induced preterm labor [58], as well as in modulating immune responses during these processes and with vaccination.

## Supporting Information

S1 Fig Purity of TFAM. A 15% SDS-PAGE gel of 2 μg of purified TFAM electrophoresed next to molecular weight standards. The predicted molecular weight of the TFAM construct used in this study is 24,667 Da.
(TIF)

S2 Fig Cellular association of CpG-B (PD). Representative flow cytometry histogram of FAM-labeled CpG-B (PD) staining on CD19^+^ B cells in PBMCs after 1 hour incubation with CpG-B (PD) alone (0.08 μM, black line), CpG-B (PD) (0.08 μM) + TFAM (0.325 μM, dotted line) or media control (gray filled). **(A)** Median fluorescence intensity (MFI) of FAM-labeled-CpG-B (PD) staining on B cells in PBMCs (n = 3) after 1 hour of incubation with TFAM (0.325 μM). P-values are compared to CpG-B (PD) only control **(B)**.
(TIF)
