# Supplementary figures and images for: Mechanism of Mitochondrial Transcription Factor A Attenuation of CpG-Induced Antibody Production
Source: PLoS One. 2016 Jun 9;11(6):e0157157. doi: 10.1371/journal.pone.0157157 (PMC4900672; doi:10.1371/journal.pone.0157157)

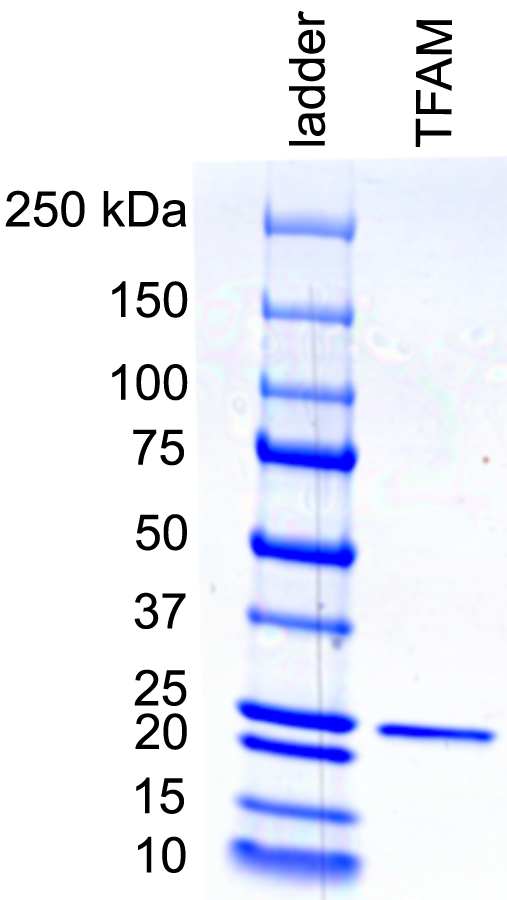

Supplement: S1 Fig — A 15% SDS-PAGE gel of 2 μg of purified TFAM electrophoresed next to molecular weight standards. The predicted molecular weight of the TFAM construct used in this study is 24,667 Da. (TIF) [file pone.0157157.s001.tif]

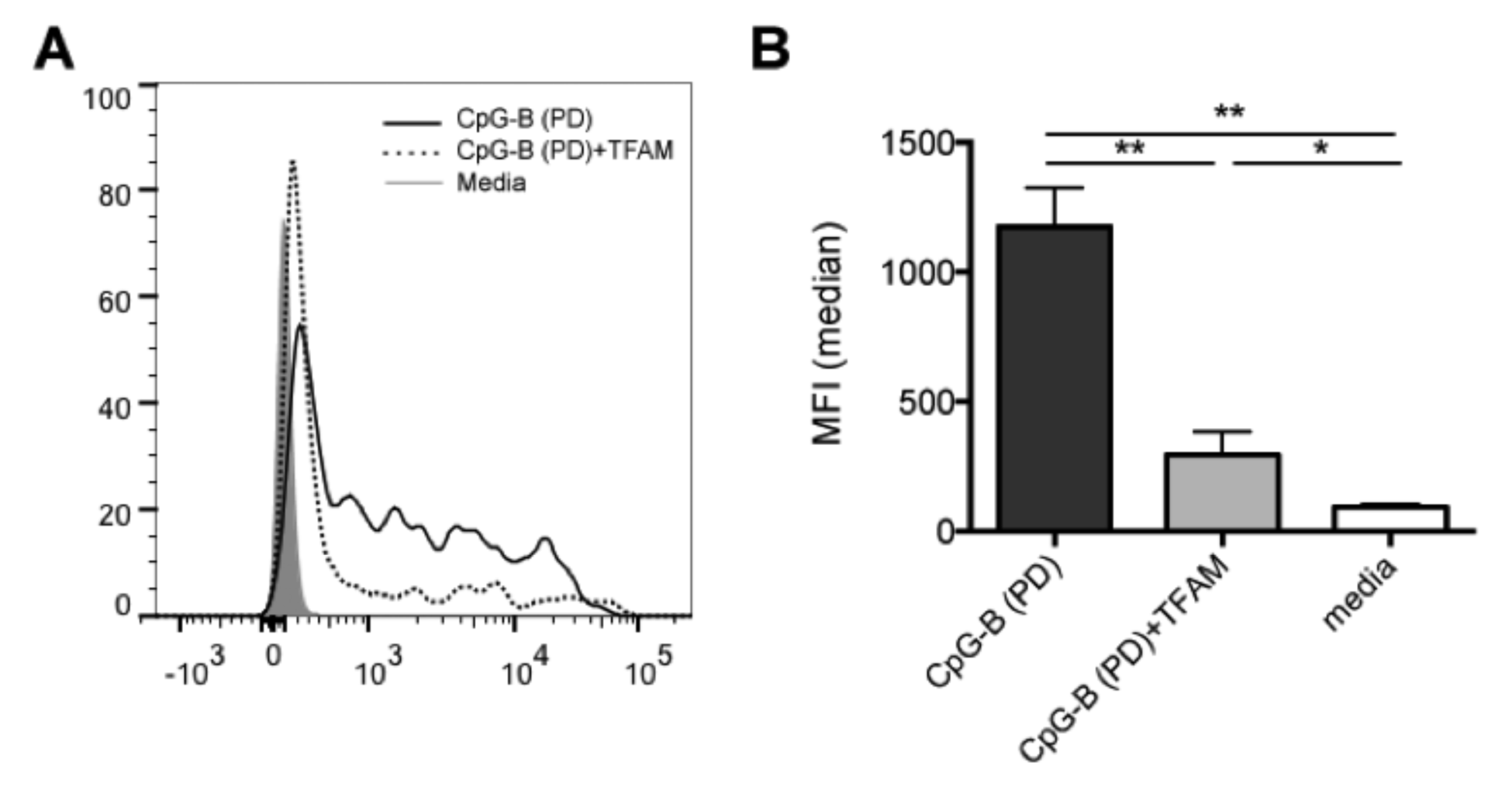

Supplement: S2 Fig — Representative flow cytometry histogram of FAM-labeled CpG-B (PD) staining on CD19+ B cells in PBMCs after 1 hour incubation with CpG-B (PD) alone (0.08 μM, black line), CpG-B (PD) (0.08 μM) + TFAM (0.325 μM, dotted line) or media control (gray filled). (A) Median fluorescence intensity (MFI) of FAM-labeled-CpG-B (PD) staining on B cells in PBMCs (n = 3) after 1 hour of incubation with TFAM (0.325 μM). P-values are compared to CpG-B (PD) only control (B). (TIF) [file pone.0157157.s002.tif]
